# Dr. Charles E. Bennett

**Published:** 1917-02

**Authors:** 


**Dr. Charles E. Bennett,** Syracuse 1880, of Cortland, died at the Cortland Hospital, Nov. 30, of septicaemia, aged 58.

